# Comprehensive analysis of metabolic pathway activity subtypes derived prognostic signature in hepatocellular carcinoma

**DOI:** 10.1002/cam4.4858

**Published:** 2022-06-01

**Authors:** Junyu Huo, Jinzhen Cai, Liqun Wu

**Affiliations:** ^1^ Liver Disease Center The Affiliated Hospital of Qingdao University Qingdao China

**Keywords:** hepatocellular carcinoma, metabolism, prognostic, signature, subtype

## Abstract

**Objective:**

Metabolic reprogramming is one of the hallmarks of cancer, but metabolic pathway activity‐related subtypes of hepatocellular carcinoma (HCC) have not been identified.

**Methods:**

Based on the quantification results of 41 metabolic pathway activities by gene set variation analysis, the training cohort (*n* = 609, merged by TCGA and GSE14520) was clustered into three subtypes (C1, C2, and C3) with the nonnegative matrix factorization method. Totally 1371 differentially expressed genes among C1, C2, and C3 were identified, and an 8‐gene risk score was established by univariable Cox regression analysis, least absolute shrinkage and selection operator method, and multivariable Cox regression analysis.

**Results:**

C1 had the strongest metabolic activity, good prognosis, the highest CTNNB1 mutation rate, with massive infiltration of eosinophils and natural killer cells. C2 had the weakest metabolic activity, poor prognosis, was younger, was inclined to vascular invasion and advanced stage, had the highest TP53 mutation rate, exhibited a higher expression level of immune checkpoints, accompanied by massive infiltration of regulatory T cells. C3 had moderate metabolic activity and prognosis, the highest LRP1B mutation rate, and a higher infiltration level of neutrophils and macrophages. Internal cohorts (TCGA, *n* = 370; GSE14520, *n* = 239), external cohorts (ICGC, *n* = 231; GSE116174, *n* = 64), and clinical subgroup validation showed that the risk score was applicable for patients with diverse clinical features and was effective in predicting the prognosis and malignant progression of patients with HCC. Compared with the low‐risk group, the high‐risk group had a poor prognosis, enhanced cancer stem cell characteristics, activated DNA damage repair, weakened metabolic activity, cytolytic activity, and interferon response.

**Conclusion:**

We identified HCC subtypes from the perspective of metabolism‐related pathway activity and proposed a robust prognostic signature for HCC.

## Graphical abstract

Metabolic reprogramming is one of the hallmarks of cancer, but metabolic pathway activity related subtype of hepatocellular carcinoma(HCC) has not been identified. In our work, we found for the first time that HCC could be clustered into three different subtypes according to the activity of metabolic pathways.

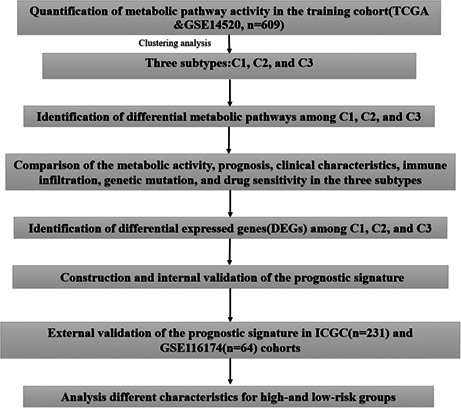

## 1 BACKGROUND

As tumors progress, they develop metabolic patterns that are significantly different from those of normal cells to feed their rapid growth; for example, even in the presence of normoxia, tumor cells preferentially use glycolysis, a phenomenon scientists describe as metabolic reprogramming,^1^ which has been summarized as one of the 10 characteristics of cancer.^2^ Increasing evidence indicates that metabolic abnormalities not only lead to the metabolic reprogramming of tumor cells but also remodel the microenvironment through different metabolic signaling pathways, thus synergistically promoting the occurrence and development of tumors.^3^, ^4^, ^5^ With a better understanding of the complexity of tumor metabolism, people have realized that the metabolism of tumor cells of different subtypes of the same tumor is different, the metabolic phenotype and metabolic dependence are changing in different stages of cancer development, and there is metabolic heterogeneity among different tumors, which leads to the difference in the efficacy of metabolic drugs in different tumors.^6^, ^7^ Therefore, developing personalized treatment strategies for cancer patients according to metabolic heterogeneity is becoming increasingly important. An in‐depth understanding of the molecular mechanism of metabolic regulation of cancer is of great significance for improving treatment effects and reducing the incidence and mortality of cancer.

Globally, hepatocellular carcinoma (HCC) is one of the most commonly seen malignancies, with high prevalence and death rate,^8^ and is also the primary hepatic malignant tumor with the highest incidence.^9^ Due to the characteristics of rapid progression and early metastasis of HCC, early diagnosis is difficult in clinical practice.^10^ HCC has a low resection rate, a high degree of malignancy, and is prone to recurrence after surgery^11^; it is not susceptible to radiation therapy and chemotherapy and lacks effective targeting and immunotherapy, resulting in a poor prognosis.^12^ The liver is the main metabolic organ of the human body, which undertakes important functions. Such as biotransformation, synthesis and detoxification, and the onset and progression of HCC is often accompanied by intricate and diversified metabolic abnormalities. Previous studies have shown that the changes in metabolism‐related proteins were the largest difference between liver cancer and non‐liver cancer tissues.^13^ After that, some scholars classified HCC from a molecular perspective based on the expression of metabolism‐related genes, which was used to judge the prognosis of patients with HCC.^14^ Currently, a number of reports have indicated that the metabolic remodeling activity exhibited by HCC promotes the progression of cancer. Recent studies have shown that the activation of the fatty acid metabolism signaling axis plays a critical role in the malignant transformation of nonalcoholic steatohepatitis and is a vital factor in the formation and maintenance of initiating cells of HCC.^15^ Previous studies have found that under nutrient deficiency, HCC cells can use ketone bodies for energy to maintain their own survival and proliferation.^16^ Researchers have found that HCC cells lost the gluconeogenic activity of normal liver cells and have switched cancer cell metabolism from glycolysis back to gluconeogenesis with dexamethasone, yielding promising therapeutic outcomes.^17^ Another research team used multi‐omics technology to detect changes in the transcriptome and metabolomics during the process of HCC formation and liver tissue regeneration and found that the gradual loss of branched‐chain amino acid catabolism promotes the occurrence and development of HCC and can independently predict clinical outcomes.^18^ Therefore, it is urgent to elucidate the metabolic mechanism of HCC and to draw more precise gene and molecular maps to provide a theoretical basis for identifying new therapeutic targets and realizing personalized therapy.

In this study, we attempted to carry out cluster analyses on HCC specimens by quantifying metabolic pathway activity and observed whether there were diversities in clinical outcomes among diverse metabolic subtypes. We also sought to identify driver genes that form different metabolic subtypes, striving to help clinical oncologists adapt treatment strategies to the specific tumor metabolism of patients with HCC.

## 2 MATERIALS AND METHODS

### 2.1 Data acquisition

Four separate HCC cohorts with intact genetic expression profiles and clinical information were enrolled in our work: The Cancer Genome Atlas (TCGA, https://portal.gdc.cancer.gov/) cohort included 370 patients with prognostic information; the GSE14520 (GEO, https://www.ncbi.nlm.nih.gov/geo/) cohort involved 239 patients with prognostic information; the International Cancer Genomics Consortium (ICGC, https://dcc.icgc.org/releases/current/Projects/LIRI‐JP) cohort involved 231 patients with prognostic information; and the GSE116174(GEO, https://www.ncbi.nlm.nih.gov/geo/) cohort involved 64 patients with prognostic information. The clinicopathologic data for all cohorts are displayed in Table 1. To ensure the accuracy of the analysis outcomes, the genetic expression profiles of the above four independent cohorts were uniformly adjusted to the transcripts per million kilobase (TPM) format using the R package “limma”, and the ComBat function of the R “SVA” package was employed to remove batch effects between diverse datasets.^19^, ^20^ During the data collection process, we strictly abided by the access rules of the publicly available database, and the acceptance of the regional ethics board was not needed due to the data being collected from a public database.

**TABLE 1:** Clinicopathological information for all cohorts

	TCGA	GSE14520	ICGC	GSE116174
Survival status				
Alive	240	143	189	37
Dead	130	96	42	27
Gender				
Male	249	189	170	6
Female	121	28	61	58
Age				
≤65	232	198	89	55
>65	138	19	142	9
Cirrhosis				
None		17		
Yes		200		
Grade				
G1	55			
G2	177			
G3	121			
G4	12			
AJCC TNM stage				
I&II	256	168	141	53
III&IV	90	49	90	11
AFP				
≤300 ng/ml	197	120		
>300 ng/ml	62	97		
Vascular invasion				
None	206			
Micro & macro	108			
BCLC stage				
0‐A		165		
B‐C		52		
CLIP_Score				
<2		169		
≥2		48		
ALT				
≤50 U/L		127		
>50 U/L		90		

### 2.2 Quantification and cluster analysis of metabolic pathway activity

We extracted a total of 41 KEGG metabolic pathways from the molecular signature database (MSigDB, http://www.gsea‐msigdb.org/gsea) (Table S1) and used the enrichment score (ES) generated from the gene set variation analysis (GSVA) to quantify the activity of metabolic pathways.^21^ The higher the value of ES is, the stronger the activity of the metabolic pathway. In this way, we convert the pathway activity of the sample into a matrix. Subsequently, we merged TCGA and GSE14520 into a training set and applied the R package “NMF” to complete nonsupervised hierarchic clustering analyses on the training set to identify HCC metabolic activity subtypes.^22^ The R package “factoextra” was employed to identify the optimum number of clusters (k) and to visualize the clustering results.^23^ The silhouette width of different subtypes was drawn by the R package “cancersubtypes.” The score of each sample silhouette width was between −1 and 1, and the closer to 1 the score was, the more accurate the sample classification.^24^

### 2.3 Exploration of the characteristics of different metabolic subtypes

First, we applied the R package “limma” to perform a differential analysis of the results of the GSVA to find metabolic pathways with significant differences among different metabolic subtypes, and fdr <0.05 indicated statistical significance.^25^ The Kaplan–Meier (K–M) approach and log‐rank test were leveraged to identify diverse metabolic subtypes and differential metabolic pathways with prognostic differences. Single‐sample gene set enrichment analysis (ssGSEA) was employed to evaluate the degree of tumor immunocyte infiltration.^26^ We also downloaded somatic mutation information from TCGA and mapped the somatic mutation landscape of different metabolic subtypes using the R package “maftools.”

### 2.4 Establishment and verification of a prognosis‐related signature

We first applied the R package “limma” to select the differentially expressed genes (DEGs) among different metabolic subtypes (*p* < 0.001) and utilized the R package “clusterProfiler” to complete KEGG and Gene Ontology (GO) term enrichment analysis on the intersecting differential genes. Genes related to OS in the learning cohort (*n* = 609) were identified using univariable Cox regression analyses with screening *p* values set to <0.05.^27^ Overfitting between prognostic genes was excluded by LASSO arithmetic to decrease the prognostic genes, and a punishment parameter modification was utilized by 10‐fold cross‐verification on the foundation of the R package “glmnet.”^28^ The genes with nonzero regression coefficients acquired from LASSO regression analyses were involved in the multivariable Cox regression analyses.^29^ The risk scoring was identified by multiplying the expression level of every gene by the relevant regressive coefficient from the multivariable Cox regression analyses of every gene.^30^ Separating patients into the risk_high_ group and risk_low_ group on the foundation of the mid‐value of risk scoring.^31^ Two independent cohorts, ICGC (*n* = 231) and GSE116174 (*n* = 64), were utilized for external verification of the prognostic signature.

### 2.5 Exploration of the characteristics of diverse risk groups

We used an innovative one‐class logistic regression (OCLR) machine‐learning algorithm to calculate cancer stem cell indices (mRNAsi) for the quantification of the stemness of tumor samples.^32^ The value of mRNAsi ranges from 0 to 1, and the higher the value is, the higher the similarity between tumor cells and stem cells. The ESTIMATE (Estimation of STromal and Immune cells in MAlignant Tumor tissues using Expression data) algorithm calculated the stromal score (that captures the presence of stroma in tumor tissue) by analyzing the specific gene expression characteristics of stromal cells to quantified the stromal components in the tumor microenvironment (TME).^33^ ssGSEA was utilized to quantify the immune function in diverse risk groups. Visualization of the gene mutation landscape for diverse risk groups was done via the R package “maftools.” The R package “pRRophetic” was employed to study the drug susceptibility of diverse risk groups. GSEA was used to reveal the underlying molecular mechanisms of different risk groups.

### 2.6 Statistical analysis

All statistical analyses were accomplished with R software (v3.6.3). Student's *t*‐ test was performed for continuous variables with a normal distribution, analysis of variance (ANOVA) was used for multiple group comparisons. Pearson chi‐square test was used to compare categorical variables. Spearman's correlation analyses were utilized to assess the association between continuous variables. The K–M method with a two‐sided log‐rank test was employed to compare the OS of patients between subgroups. Time‐dependent receiver operating characteristic curves were used to evaluate the prediction accuracy of the prognostic indicators.^34^ A two‐sided *p* value <0.05 was considered statistically significant.

## 3 RESULTS

### 3.1 Different metabolic pathway subtypes with different survival outcomes

**FIGURE 1:**
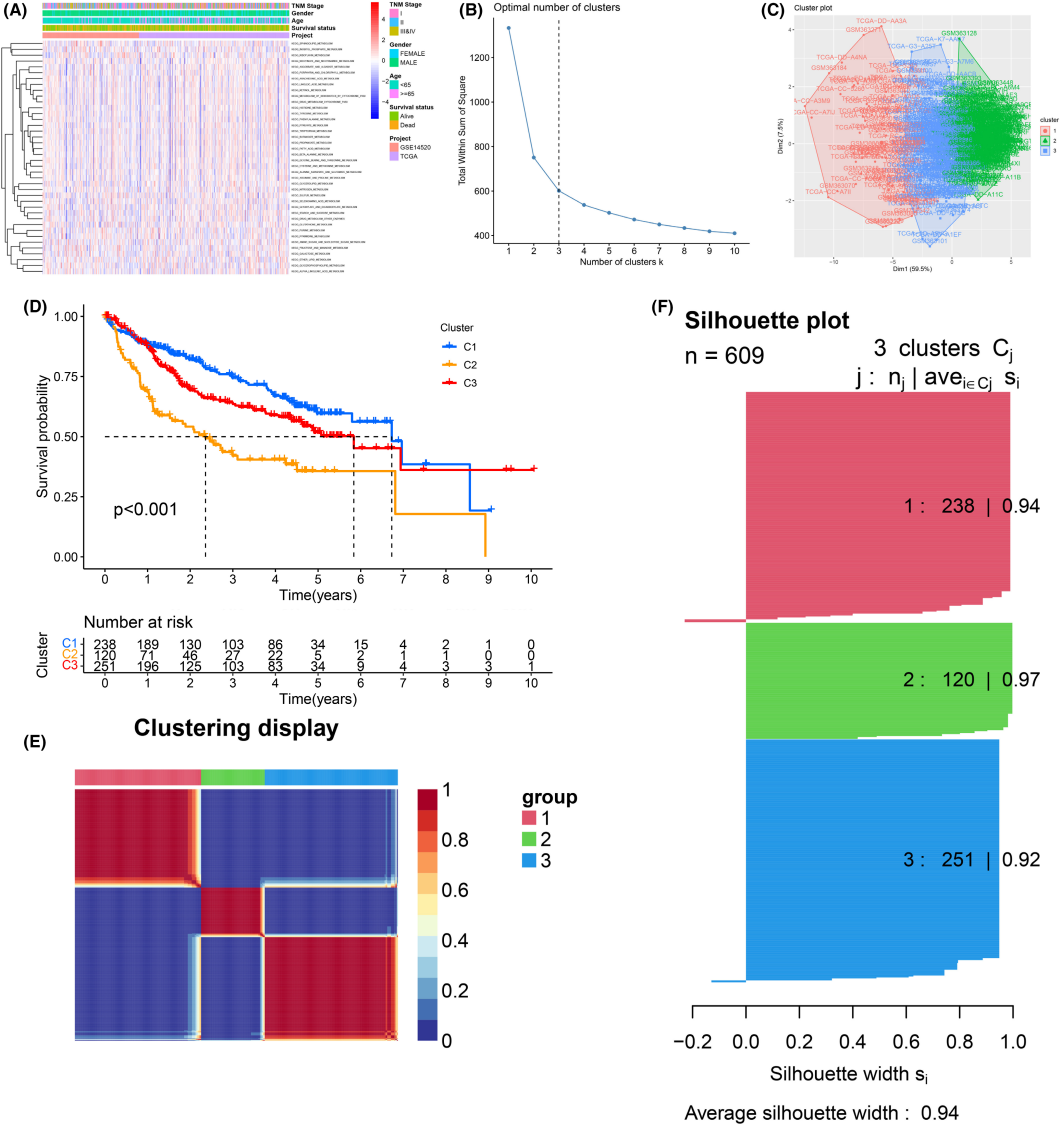
Metabolic pathway activity clustering analysis. (A) Heatmap of 41 KEGG metabolic pathways activity. (B) Optimal number of clusters. (C) Cluster plot. (D) K–M curve. (E) Clustering display heatmap. (F) Silhouette plot.

The ES of 41 metabolism‐related pathways was calculated by the GSVA according to the gene expression profiles of the training sets (Figure 1A). Based on the optimal number of clusters (K) determined by the R package “factoextra,” the training cohort (*n* = 609, merged by TCGA and GSE14520) was clustered into three distinct subtypes (Figure 1B,C). The survival result of Cluster 1(C1) was the best, Cluster 3(C3) was the second best, and Cluster 2(C2) was the worst (Figure 1D). The clustering map displayed that there was strong consistency between samples from the identical subgroup, while there was little correlation between samples from different subgroups (Figure 1E). The average silhouette width value was 0.94, indicating the high accuracy of the results of cluster analysis (Figure 1F).

### 3.2 Characteristics of different metabolic pathway subtypes

**FIGURE 2:**
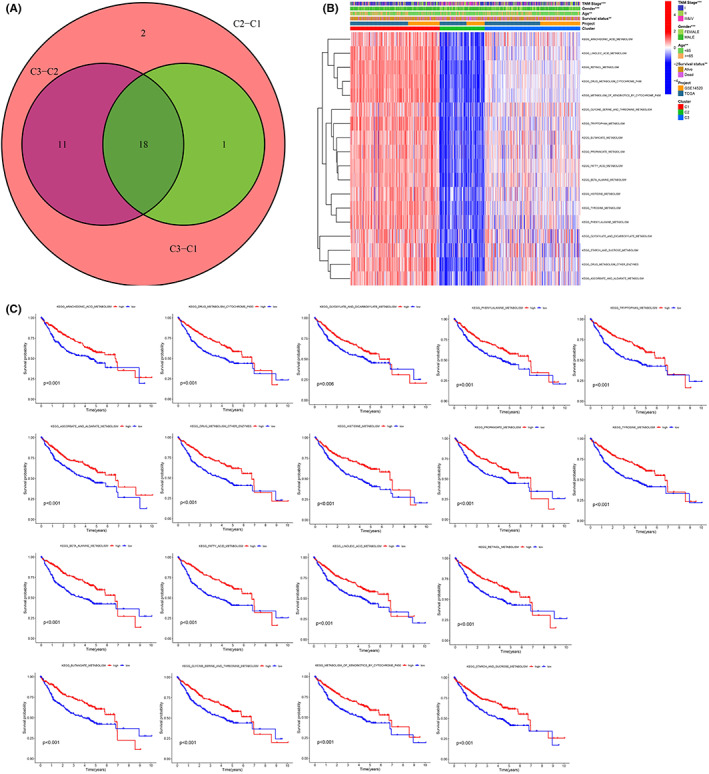
Intersecting differential metabolic pathways among the three subtypes. (A) Venn diagram. (B) Heatmap of 18 intersecting differential metabolism paths. (C) K–M survival curve of 18 intersecting differential metabolism paths.

**FIGURE 3:**
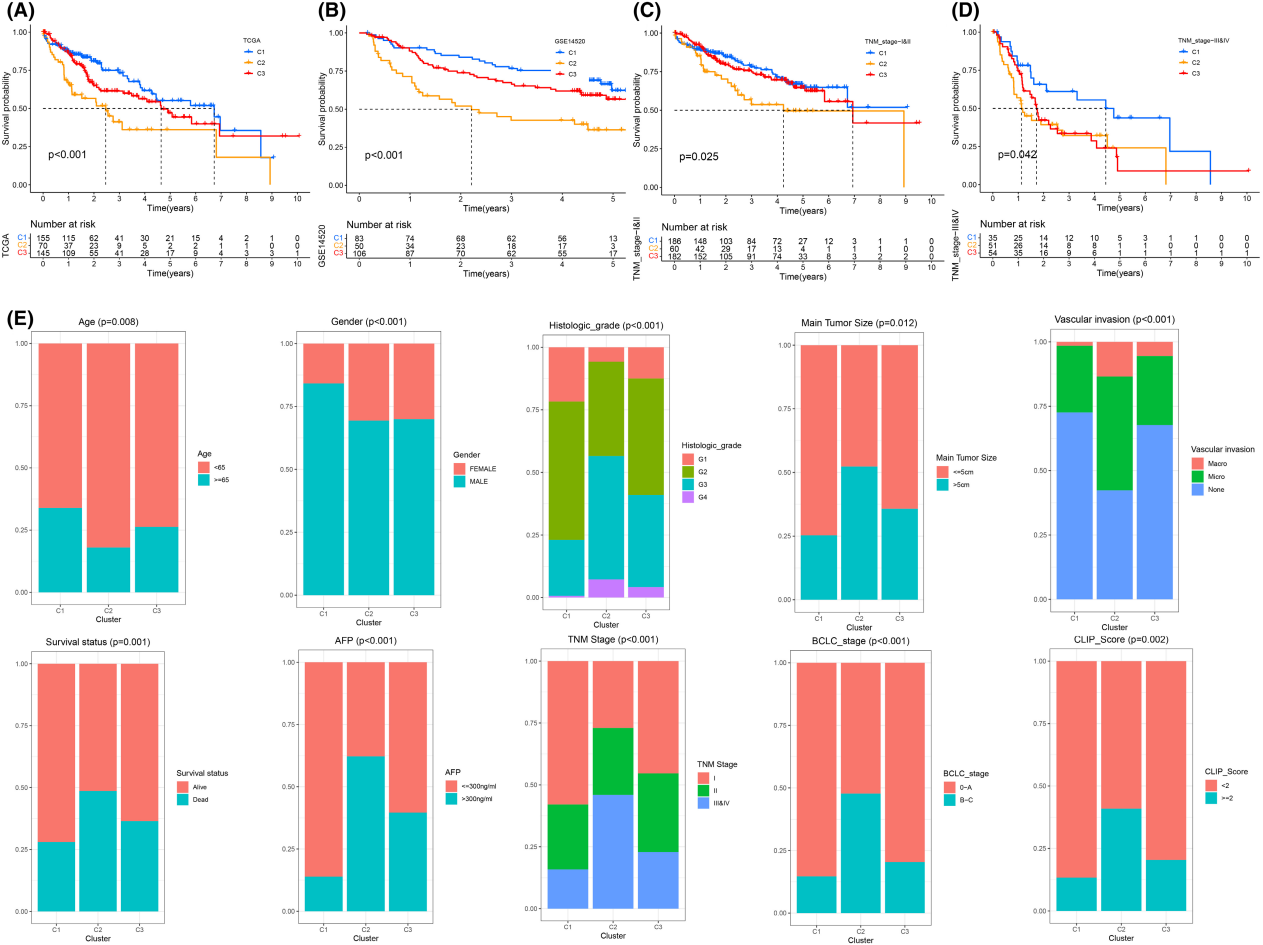
Clinical characteristics of different metabolism path subtypes. (A‐D) K–M survival curve of different clusters in TCGA, GSE1450, TNM phase I‐II, TNM phase III‐II. (E) Clinic features for different subtypes.

**FIGURE 4:**
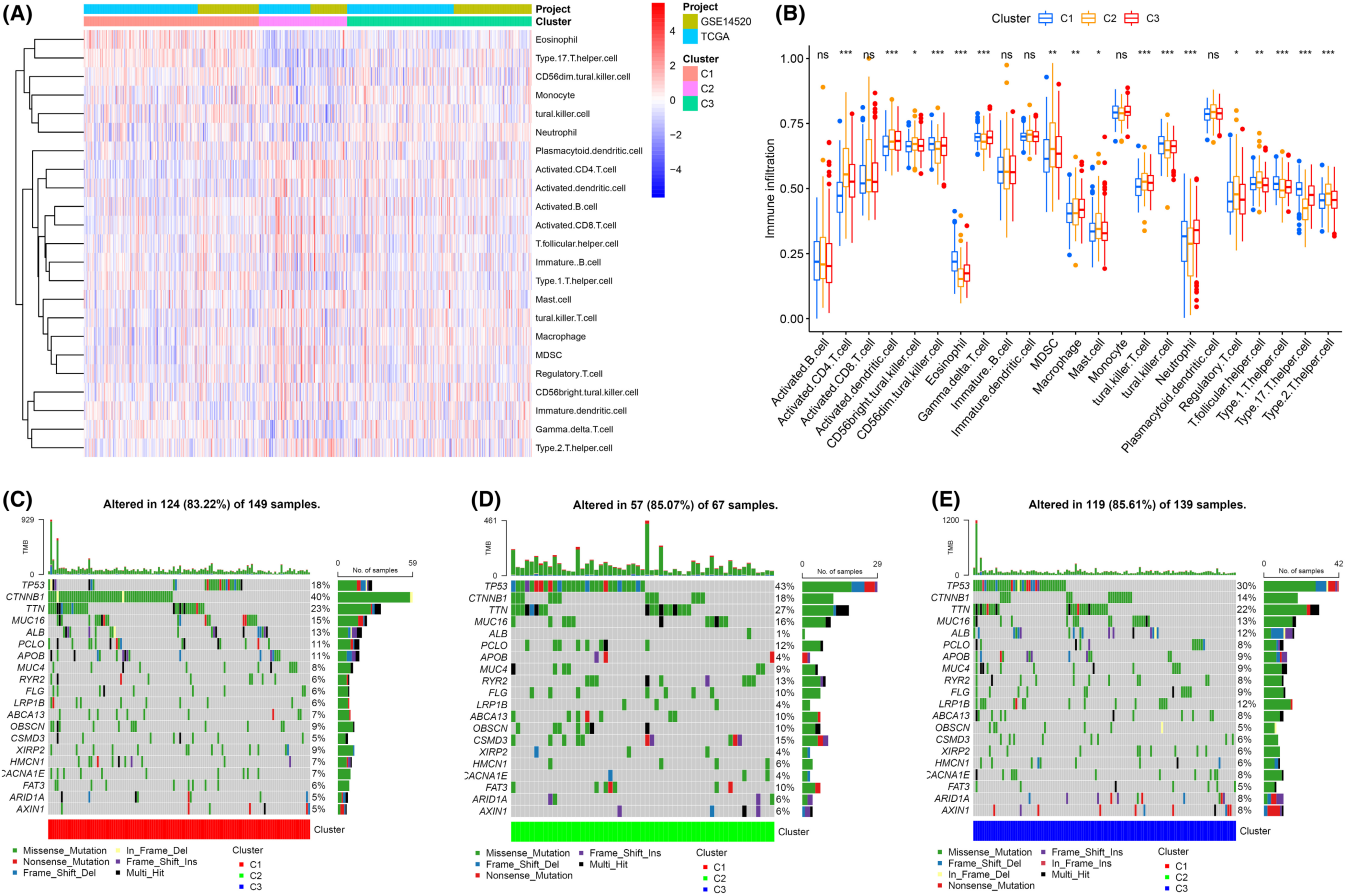
Immune infiltration and genetic mutational differences in different metabolic pathway subtypes. (A, B) Immune infiltration for different subtypes (“***”, *p* < 0.001; “**”, *p* < 0.01; “*”, *p* < 0.05). (C–E) Genetic mutational landscape for different subtypes.

**FIGURE 5:**
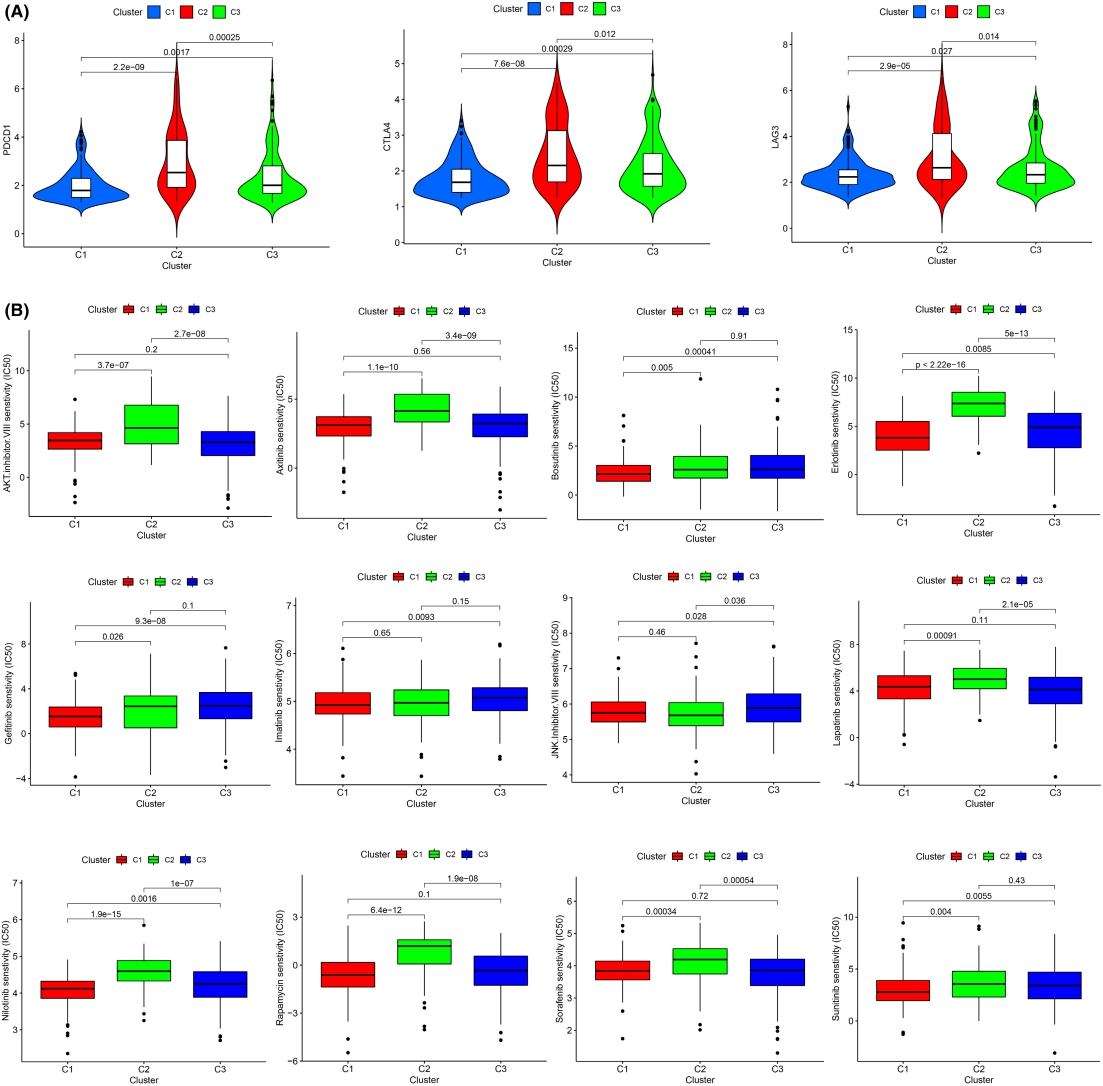
Drug sensitivity in different metabolic pathway subtypes. (A) The expression level of immune checkpoints for different subtypes. (B) The IC50 for different anticancer‐targeted drugs in different subtypes.

A total of 18 intersecting differential metabolic pathways were identified among the three different subtypes by pairwise comparison (C1 vs. C2, C1 vs. C3, and C2 vs. C3) (FDR < 0.05, Figure 2A), such as retinol metabolism, tryptophan metabolism, and fatty acid metabolism. Interestingly, according to the quantitative results (ES) of pathway activity, we found that the activity of these 18 metabolic pathways was consistent among subtypes, that is, C1 > C3 > C2 (Figure 2B). The K–M survival curve revealed that the prognosis of patients with upregulated activity of these 18 metabolic pathways was remarkably better than that of patients with downregulated activity of these 18 metabolic pathways (Figure 2C). Whether in TCGA cohort or GSE14520 cohort, the prognosis of C1 was the best, C3 was the second best, and C2 was the worst (Figure 3A,B). For patients with TNM phase I‐II, the prognosis of patients with C2 was the worst, and for patients with TNM phase III‐IV, the prognosis of patients with C1 was the best (Figure 3C,D). The clinical characteristics were different between different subtypes, for example, the proportion of patients who were alive, age ≥65 years, male, TNM stage I‐II in the C1 subtype was higher than the other two subgroups, while the proportion of patients who died, age <65 years, vascular invasion, main tumor size >5 cm, AFP >300 ng/ml, poorly differentiated histology grade (G3‐4), and advanced stage (TNM stage III–IV, BCLC phase B‐C, and CLIP scoring ≥2) in the C2 subtype was greater than the other two subtypes (Figure 3E). Remarkable diversities also existed in the infiltrating abundance of immunocytes between diverse subtypes; for example, the infiltration degree of regulatory T‐cells in the C2 subtype was significantly greater than that in the other two subtypes (Figure 4A,B), while the infiltration degree of neutrophils was remarkably lower than that in the other two subtypes(Figure 4A,B). The infiltration abundance of eosinophils and natural killer cells in the C1 subtype was remarkably greater than that in the other two subtypes (Figure 4A,B). The infiltration abundance of macrophages in the C3 subtype was remarkably greater than that in the other two subtypes (Figure 4A,B). In terms of gene mutations, the mutation frequencies of TP53, TTN, and CSMD3 in C2 were the highest, and the mutation frequencies of CTNNB1, APOB, and ALB in C1 were the highest (Figure 4C–E). The mutation rate of LRP1B in C3 was the highest among the three subtypes (Figure 4C–E). The expression trends of the three immunotherapy targets (PDCD1, CTLA4, and LAG3) were as follows: C2 > C3 > C1 (Figure 5A). In terms of targeted therapy, C1 was the most sensitive to targeted drugs, such as sorafenib, erlotinib, and nilotinib (Figure 5B). Based on the above findings, we summarized the characteristics of the three subtypes in Table 2.

**TABLE 2:** Characteristics summarization of the three metabolic pathway activity subtypes

Subtype	C1	C2	C3
Metabolic pathway activity	high	low	moderate
Prognosis	good	poor	intermediate
Clinical characteristics	age ≥65	age <65	None specific
	male	poor‐differentiated	
	well‐differentiated	main tumor size >5 cm	
	main tumor size ≤5 cm	AFP >300 ng/ml	
	AFP ≤300 ng/ml	vascular invasion	
		advanced stage	
Immune cells infiltration	Eosinophil	Regulatory.T.cell	Macrophage
	Type.1.T.helper.cell	MDSC	Neutrophil
	Type.17.T.helper.cell	Mast cell	Gamma.delta.T.cell
	natural killer cells	Activated.CD4.T.cell	
		Type.2.T.helper.cell	
		T.follicular.helper.cell	
Gene mutation	CTNNB1	TP53	LRP1B
	APOB	TTN	APOB
	ALB	CSMD3	ALB
Drug sensitivity	Sorafenib	immune checkpoint inhibitors	Sorafenib
	Sunitinib	JNK.Inhibitor.VIII	Rapamycin
	Gefitinib		Axitinib
	Bosutinib		Lapatinib
	Erlotinib		AKT.inhibitor.VIII
	Nilotinib		

### 3.3 Construction and verification of the eight‐gene prognosis risk score

**FIGURE 6:**
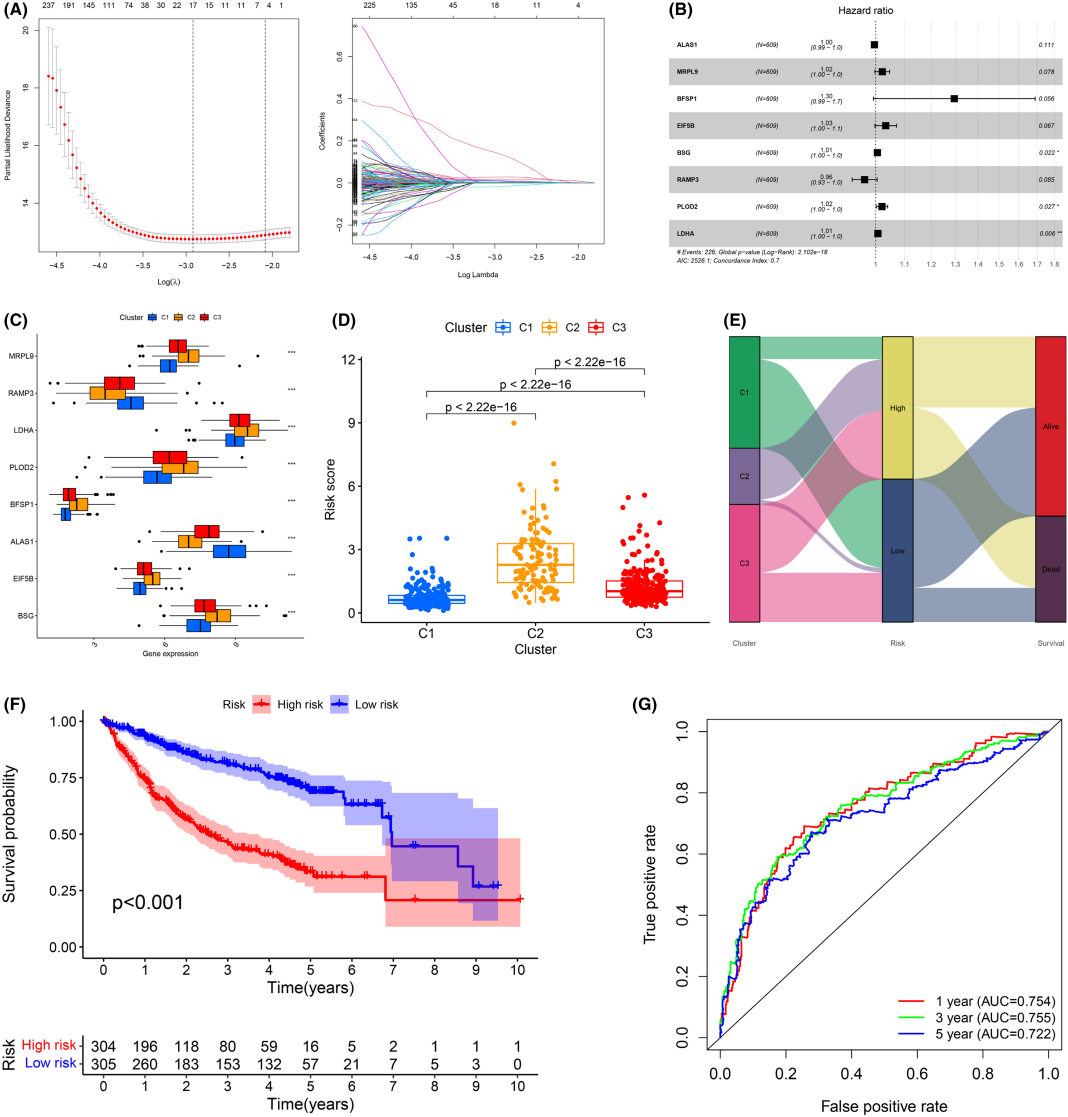
Establishment of the eight‐gene risk scoring in the learning cohort. (A, B) LASSO and multivariable Cox regression analyses. (C) The expression level of the eight genes in diverse clusters (“***”, *p* < 0.001; “**”, *p* < 0.01; “*”, *p* < 0.05). (D, E) Correlation analysis between the risk score and metabolic subtypes. (F, G) K–M and time‐reliant ROC curves.

**FIGURE 7:**
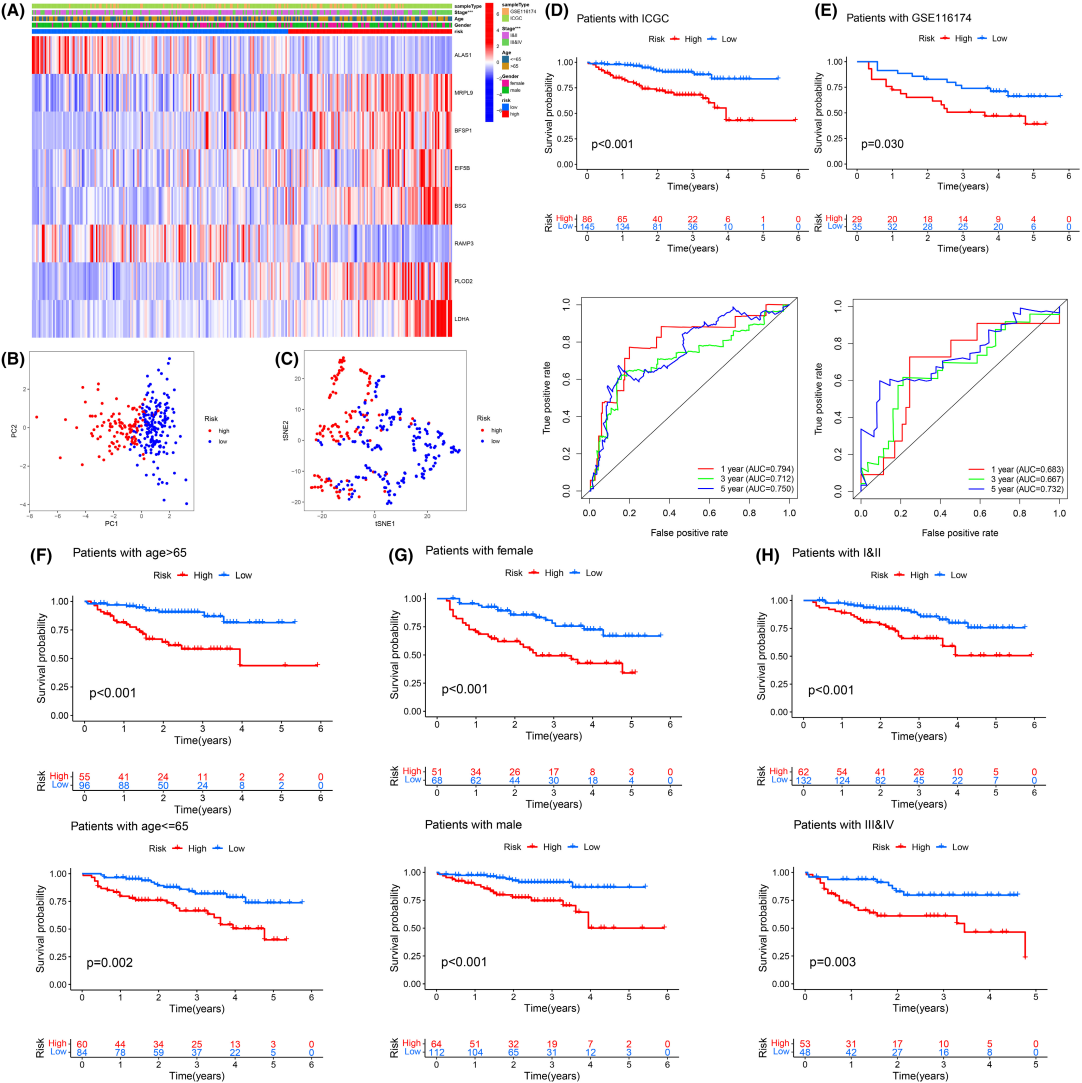
External verification of the risk scoring in the ICGC and GSE116174 cohorts. (A) The heatmap. (B) The PCA. (C) The t‐SNE analyses. (D) The K–M analyses and the time‐reliant ROC analyses for the risk scoring in forecasting the OS of patients in the ICGC cohort. (E) The K–M analyses and the time‐reliant ROC analyses for the risk scoring in forecasting the OS of patients in the GSE116174 cohort. (F) Age. (G) Gender. (H) AJCC‐TNM stage.

A total of 1371 overlapping DEGs were identified among the three different subtypes by pairwise comparison (C1 vs. C2, C1 vs.C3, and C2 vs. C3) (FDR < 0.001, Figure S1A,B). Interestingly, GO and KEGG analyses revealed that these DEGs were not only involved in metabolism but also related to COVID‐19, the cell cycle, peroxisomes, spliceosomes, etc. (Figure S1C,D). Univariate Cox regression analysis revealed that 1202 of 1371 DEGs exhibited prognostic significance (*p* < 0.05, Table S2). Seventeen genes with nonzero coefficients were retained after LASSO regression analysis (Figure 6A). Finally, an eight‐gene risk score was constructed via multivariable Cox regression analysis (Figure 6B). The formula of risk scoring was as follows: ALAS1* −0.00385+ MRPL9* 0.02187+ BFSP1* 0.2585+ EIF5B* 0.03313+ BSG* 0.005886+ RAMP3* −0.036168+ PLOD2* 0.021+ LDHA* 0.007466. The expression levels of eight genes among the three metabolic subtypes were significantly different (Figure 6C). The risk score of C2 was the highest, C3 was the second highest, and C1 was the lowest (Figure 6D). Most of the samples in C2 were categorized as risk_high_ patients, and most of the samples in C1 were categorized as risk_low_ patients (Figure 6E). As per the mid‐value of the risk score (0.925), the learning cohort was separated into the risk_high_ group and risk_low_ group. The OS of risk_high_ patients was remarkably poorer than that of risk_low_ patients (Figure 6F). The AUC results of 1‐, 3‐, and 5‐year OS forecasted by the risk scoring were 0.754, 0.755, and 0.722, respectively (Figure 6G). Clinical correlation analyses revealed that a higher risk score was associated with death, cirrhosis, vascular invasion, main tumor size >5 cm, AFP >300 ng/ml, poorly differentiated histology grade (G3‐4), and advanced stage (TNM stage III‐IV, BCLC phase B‐C, and CLIP scoring ≥2) (Figure S2). Among the eight genes, only ALAS1 and RAMP3 were upregulated in risk_low_ patients, and the remaining six genes were all upregulated in the risk_high_ group (Figure S3A). The number of surviving patients decreased with increasing risk score (Figure S3A). In TCGA cohort, the AUC results of 1‐, 3‐, and 5‐year OS forecasted by the risk scoring were 0.780, 0.767, and 0.734, respectively (Figure S3B). In the GSE14520 cohort, the AUC results of 1‐, 3‐, and 5‐year OS forecasted by the risk scoring were 0.709, 0.740, and 0.716, respectively (Figure S3C). The prognosis of high‐risk patients was poor in all clinical subgroups (Figure S4A–L). External validation displayed remarkable differences in OS and TNM staging between the risk_high_ group and risk_low_ group (Figure 7A). Principal component analyses (PCA) and t‐distributed stochastic neighbor embedding (t‐SNE) analyses revealed that risk_high_ patients could be unequivocally distinguished from risk_low_ patients by our risk classification system (Figure 7B,C). The risk_high_ patients exhibited obviously unfavorable survival outcomes relative to the risk_low_ patients in the two separate cohorts (Figure 7D,E). In the ICGC cohort, the AUC results of 1‐, 3‐, and 5‐year OS forecasted by the risk scoring were 0.794, 0.712, and 0.750, respectively (Figure 7D). In the GSE116174 cohort, the AUC results of 1‐, 3‐, and 5‐year OS forecasted by the risk scoring were 0.683, 0.667, and 0.732, respectively (Figure 7E). The OS of risk_high_ patients was remarkably reduced regardless of sex, age, and TNM stage (Figure 7F–H). Finally, we performed a pooled analysis of the included samples to compare the performance of the risk score and the existing methods that predict the survival of patients with HCC in the given periods. By comparing AUC values, we found that the prediction performance of the risk score for 1‐year (0.755 vs. 0.645), 3‐year (0.738 vs. 0.609), and 5‐year (0.712 vs. 0.638) OS of patients with HCC was superior to the traditional AJCC TNM stage (Figure S5).

### 3.4 Characteristics of different risk groups

The stem cell index in risk_high_ patients was remarkably greater than that in risk_low_ patients, and the risk score was positively related to the stem cell index (Figure S6A). The prognoses of patients with a higher stem cell index were poor (Figure S6B). The stromal score in risk_high_ patients was remarkably smaller versus risk_low_ patients, and the risk scoring was related to the stromal scoring in a negative manner (Figure S6C). The prognosis of patients with low stromal score was poor (Figure S6D). Regarding immune function, the activities of cytolytic, type I IFN reaction, and type II IFN reaction in risk_low_ patients were significantly enhanced versus risk_high_ patients (Figure S6E,F). The mutation rate of TP53 in risk_high_ patients was remarkably greater than that in risk_low_ patients, whereas the mutation rate of CTNNB1 was remarkably smaller than that in risk_low_ patients (Figure S6G). The expression levels of multiple immune checkpoints, such as CTLA4, TIGIT, PDCD1, and LAG3, were all upregulated in the risk_high_ patients (Figure S7A). The IC50 values of multiple anticancer‐targeted drugs, such as sorafenib, rapamycin, gefitinib, and axitinib, in the risk_low_ patients were all remarkably lower than those in the risk_high_ patients (Figure S7B). GSEA showed that the pathways related to the cellular cycle, DNA damage repair, spliceosome, and RNA decomposition were abnormally activated in the risk_high_ patients (Figure S7C), while the low‐risk group maintained a higher metabolic activity, such as aliphatic acid metabolic and drug metabolic processes (Figure S7D).

## 4 DISCUSSION

In our work, we found for the first time that HCC could be clustered into three different subtypes according to the activity of metabolic pathways. Each subtype not only differs significantly in survival outcomes and metabolic activity but also has its own unique clinical features, immune microenvironment, and genetic mutational landscape. Interestingly, there were 18 intersecting differential metabolic pathways were identified among the three subtypes, and all of them had prognostic significance. Because of this, these 18 metabolic pathways were also considered to be the hub pathways for driven the formation of the three subtypes. According to the degree of activation of these 18 metabolic pathways, C1, C2, and C3 were defined as metabolism‐high, metabolism‐low, and metabolism‐moderate subtypes, respectively. C1 was the subtype with the strongest metabolic activity and the best prognosis, tending to be early stage, male and age >65 years, accompanied by infiltration with large numbers of eosinophils, natural killer cells, T‐helper 1 (Th1) cells, and Th17 cells. Its TP53 mutation rate was the lowest among the three subtypes, and the CTNNB1, APOB, and ALB mutation rates were the highest among the three subtypes. C2 was the subtype with the weakest metabolic activity and the worst prognosis, which tends to be vascular invasion, poorly differentiated tumor, higher AFP level (>300 ng/ml), advanced stage, and age <65 years), with massive infiltrative events of activated CD4 T cells, mast cells, regulatory T cells (Tregs), and Th2 cells. It is TP53, TTN, and CSMD3 mutation rates were the highest among the three subtypes, and the APOB and ALB mutation rates were the lowest among the three subtypes. The metabolic activity of C3 was moderate, the prognosis was between C1 and C2, and the infiltration level of neutrophils and macrophages was high. Its LRP1B mutation rate was the highest among the three subtypes. In addition, the potential treatment strategies for different subtypes were also different. We observed that the expression levels of the three immune therapy targets PDCD1, CTLA4, and LAG3 were significantly increased in the C2 subtype, indicating that the C2 subtype was more likely to benefit from the application of immune checkpoint inhibitors. The other two subtypes with better prognoses were more sensitive to targeted drugs, such as sorafenib, lapatinib, and rapamycin. Therefore, the redefinition of HCC subtypes from the perspective of metabolic pathway activity has enabled us to obtain an updated recognition of the heterogeneity of HCC.

Integrated analysis of transcriptome data showed that there were 1371 overlapping DEGs among the three different subtypes. These DEGs were widely involved in different biological processes in organisms, such as the cellular cycle, small molecule catabolism, and protein targeting, and were also related to COVID‐19. Considering that these 1371 overlapping DEGs may not only drive the formation of HCC metabolic subtypes, but also speculated to be an important factor affecting the prognosis of different subtypes, it is worth exploring the prognostic value of the 1371 DEGs. To clarify the prognostic significance of these DEGs for HCC, we constructed a risk score using various statistical methods. Internal and external validation showed that the risk scoring was applicable for patients with diverse clinical traits and was effective in classifying patients with HCC into two subgroups with distinct prognoses: risk_high_ and risk_low_. The risk scoring of the C2 subtype, with the worst prognostic results, was remarkably greater than that of the other two subtypes. The risk scoring of the C1 subtype, with optimal prognostic results, was remarkably smaller than that of the other two subtypes. As expected, the vast majority of patients in the C2 subtype were categorized as the risk_high_ group, while most patients in the C1 subtype were categorized as the risk_low_ group. The AUC results for the risk scoring predicting OS at 1, 3, and 5 years were >0.66 in the four separate cohorts, reflecting the accurate prediction accuracy of the risk score. The cancer stemness index is a quantitative indicator that represents the resemblance between oncocytes and stem cells, and its value ranges from 0 to 1. When it is 0, it means lower similarity with stem cells, while when it is 1, it means higher similarity with stem cells.^32^ The stromal score is a quantitative indicator representing the quantity of stromal cells in the tumor microenvironment (TME), and the higher the value is, the higher the abundance of stromal cells in the TME.^35^ The OS of the patients with a higher cancer stem cell index decreased significantly, and the cancer stem cell index of the risk_high_ patients was remarkably higher than that of the risk_low_ patients, which was closely related to the positive association between the risk scoring and the cancer stem cell index. The OS of the patients with lower stromal scoring decreased significantly, and the stromal scoring of the risk_high_ patients was remarkably smaller than that of the risk_low_ patients, which was closely related to the negative association between the risk scoring and stromal scoring. A higher risk score means a higher resemblance between oncocytes and stem cells, that is, the stronger the ability of tumor proliferation, while the lower the quantity of stroma cells in the TME, that is, oncocytes take up a larger percentage in the TME. It is conceivable that, when the risk score increases, cancer proliferation will accelerate as well, and the number of tumor cells will naturally increase and occupy a large percentage of the TME. Therefore, these two phenomena were mutually supportive. The risk score of patients with HCC who died, had cirrhosis, vascular invasion, main tumor size >5 cm, AFP > 300 ng/ml, poorly differentiated histology grade (G3‐4), and late period phase (TNM phase III‐IV, BCLC phase B‐C, and CLIP score ≥2) increased significantly, which further confirmed that the risk score promoted HCC development toward malignancy.

In recent years, molecular‐targeted therapy for HCC has made progress.^36^ Sorafenib, lenvatinib, and regorafenib have been approved for first‐ and second‐line treatment of HCC.^37^ At the same time, inhibitors targeting immune checkpoints, including anti‐CTLA‐4, PD‐1, and PD‐L1 antisubstances, have been accepted for treating HCC, but relevant studies have shown that their objective response rate is still very limited. Only a few patients have benefited, which may be related to the low immunogenicity or inhibitory immune microenvironment of HCC.^38^ We noticed that the expression level of immune checkpoints in risk_high_ patients was remarkably upregulated, which reveals that risk_high_ patients may benefit from immune therapy. Meanwhile, this may be the critical cause of its decreased cytolytic activity. Moreover, the high mutational rate of TP53 may also be a possible cause for the inferior prognoses of risk_high_ patients. The sensitivity of risk_low_ patients to sorafenib is remarkably stronger than that of risk_high_ patients, which may be related to its higher metabolic activity. Meanwhile, the abnormal activation of the cell cycle and DNA damage repair‐related pathways in high‐risk groups may indirectly reflect the acceleration of tumor cell proliferation.

Among the eight genes constituting the risk score, only ALAS1 and RAMP3 were upregulated in risk_low_ patients, and the other six genes were upregulated in risk_high_ patients. The effects of these eight genes on the phenotype of HCC and other cancers have been validated by experiments. Zhao^39^ found that silencing the expression of ALAS1 could inhibit the proliferative and metastatic abilities of colorectal cancer cells. Interestingly, according to our findings, ALAS1 is likely to play a negative regulatory role in the progression of HCC because its upregulated expression level is favorable for the OS of HCC. Fang^40^ discovered that the overexpression of RAMP3 could reduce the adverse effect of TP53 mutation on survival and was a favorable predictor for the prognosis of HCC. Tang^41^ found that, after MRPL9 was knocked out, the proliferative and metastatic abilities of HCC cells were remarkably attenuated. Gu^41^ found that the clinical outcome of HCC can be predicted according to the expression level of BFSP1, but its specific mechanism has not been clarified. Wang^42^ found that EIF5B could promote the proliferative and metastatic abilities of HCC cells via the upregulation of the expression level of ASAP1. Ma^43^ found that the overexpression of BSG could facilitate the proliferative and metastatic abilities of HCC cells. Noda^44^ found that the expression of PLOD2 was upregulated under hypoxic conditions, which was closely related to vascular invasion and intrahepatic metastasis. Sheng found that the growth and metastasis of HCC cells could be suppressed by knocking down LDHA.^45^

Based on our findings, we deem that attention should be given to the importance of the influence of changes in metabolic pathway activity on the clinical outcome of HCC. In the future, it is expected to provide broad prospects for human beings to conquer HCC by effectively identifying the specific metabolic pattern of HCC and developing corresponding individualized treatment schemes.

## 5 CONCLUSION

We identified HCC subtypes from the perspective of metabolism‐related pathway activity and proposed a robust prognostic signature for HCC.

## AUTHOR CONTRIBUTIONS

Junyu Huo and Liqun Wu designed this study, Junyu Huo collected data, analyzed the data in this study and interpreted the findings, and drafted the manuscript. Liqun Wu and Jinzhen Cai carried out data management and revised the manuscript. All authors reviewed and approved the final version of the manuscript.

## CONFLICT OF INTEREST

The authors have no conflict of interest to declare.

## ANIMAL RESEARCH (ETHICS)

Not applicable.

## CONSENT TO PARTICIPATE (ETHICS)

Not applicable.

## CONSENT TO PUBLISH (ETHICS)

Not applicable.

## PLANT REPRODUCIBILITY

Not applicable.

## Supporting information


Figure S1

Figure S2

Figure S3

Figure S4

Figure S5

Figure S6

Figure S7

Table S1

Table S2

## Data Availability

The datasets analysed for this study were obtained from The Cancer Genome Atlas (TCGA, https://portal.gdc.cancer.gov/), International Cancer Genome Consortium database (ICGC, https://dcc.icgc.org/releases/current/Projects/LIRI‐JP), Gene Expression Omnibus (GEO, https://www.ncbi.nlm.nih.gov/geo/), and the molecular signatures database (MSigDB, http://www.gsea‐msigdb.org/gsea).
