# Supplementary figures and images for: Comprehensive analysis of metabolic pathway activity subtypes derived prognostic signature in hepatocellular carcinoma
Source: Cancer Med. 2022 Jun 1;12(1):898–912. doi: 10.1002/cam4.4858 (PMC9844627; doi:10.1002/cam4.4858)

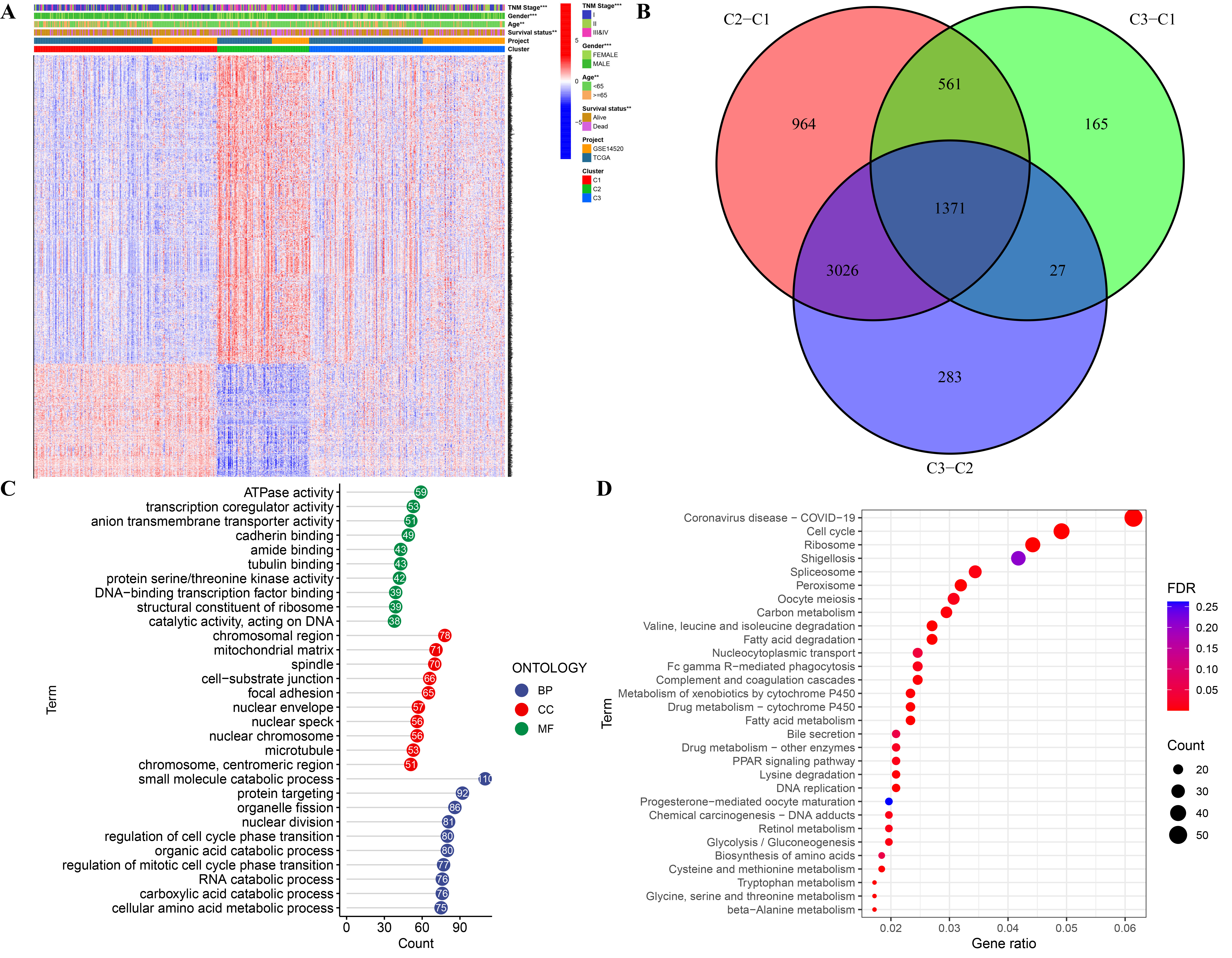

Supplement: Supplementary file 1 — Figure S1 [file CAM4-12-898-s006.jpg]

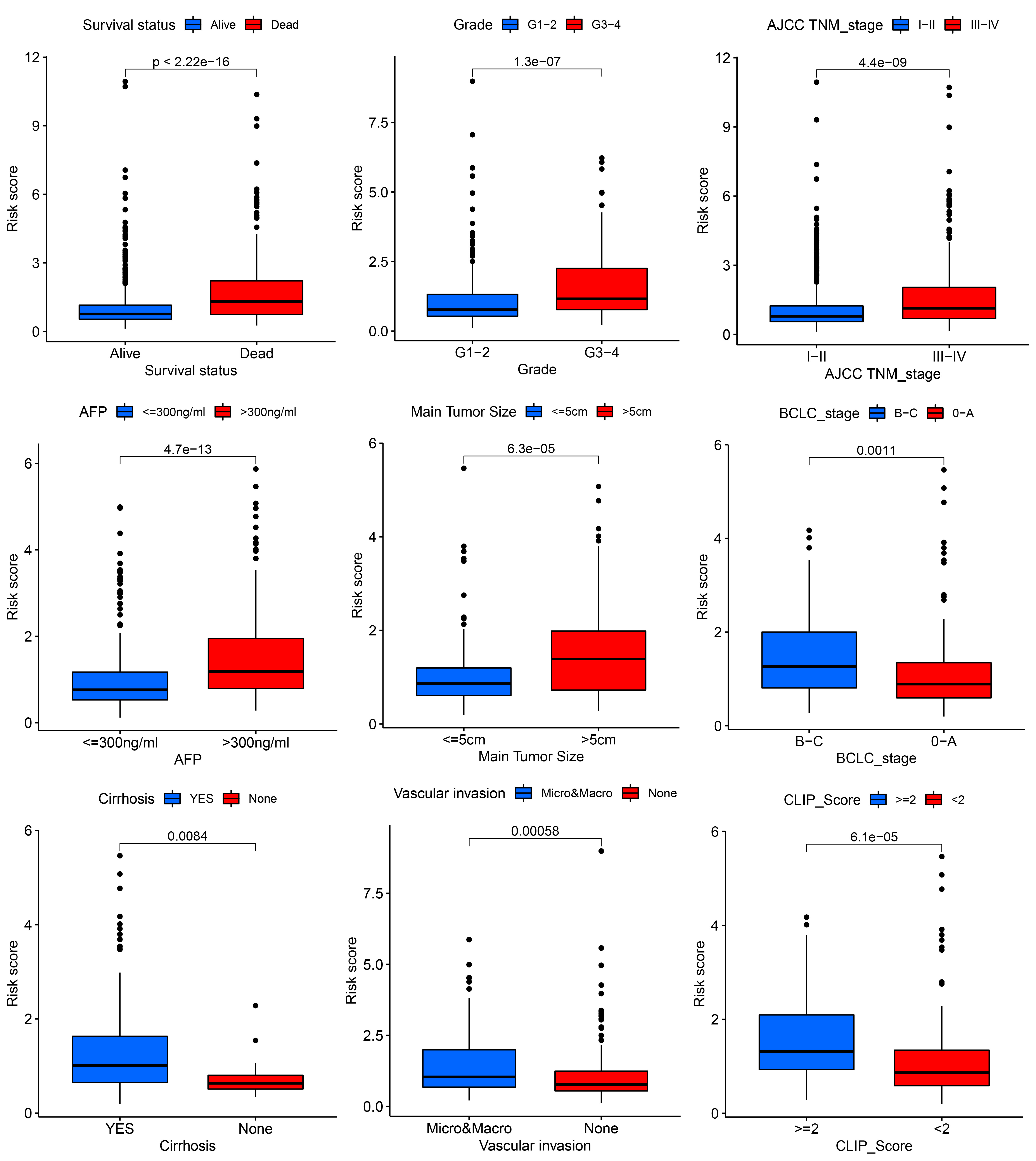

Supplement: Supplementary file 2 — Figure S2 [file CAM4-12-898-s008.jpg]

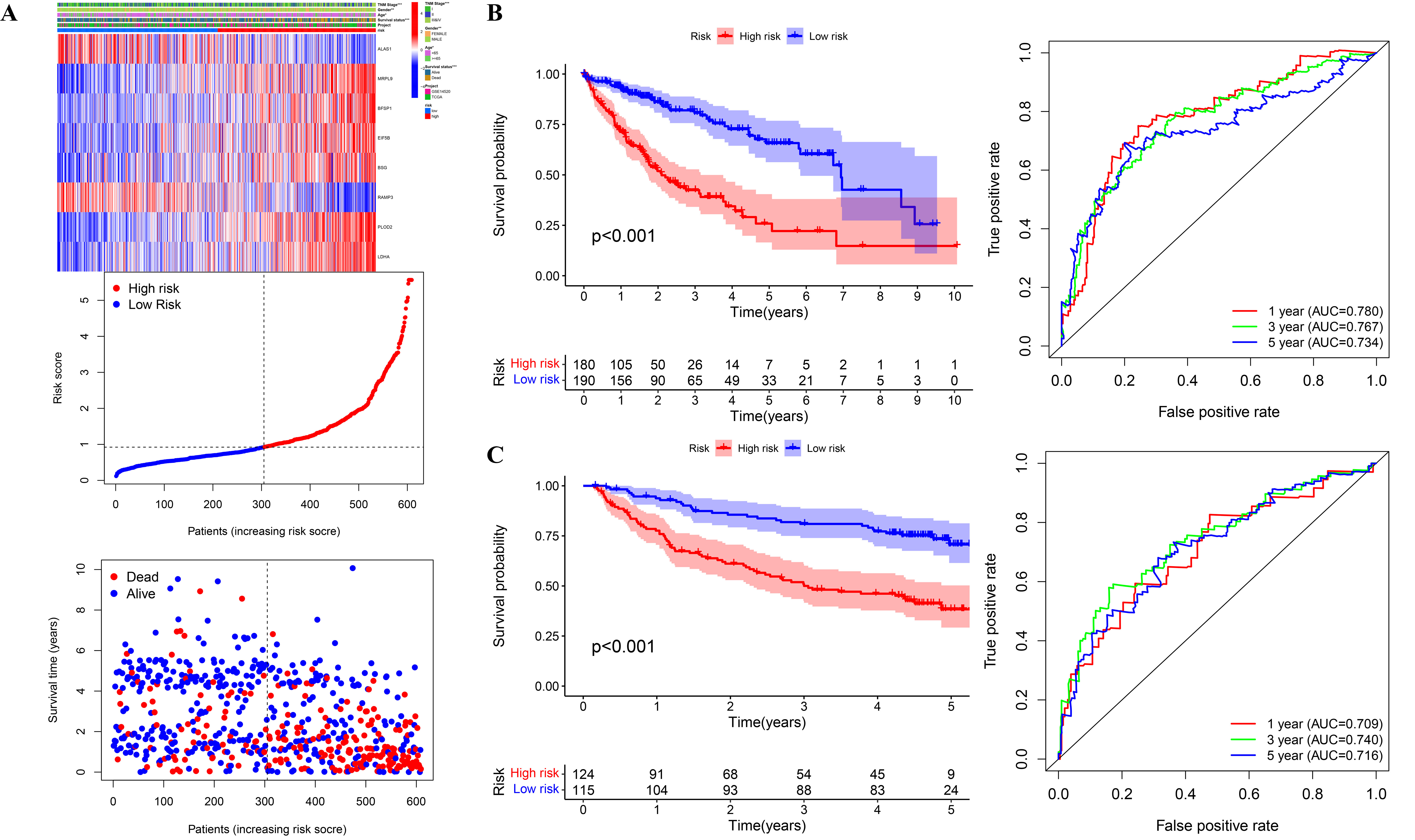

Supplement: Supplementary file 3 — Figure S3 [file CAM4-12-898-s002.jpg]

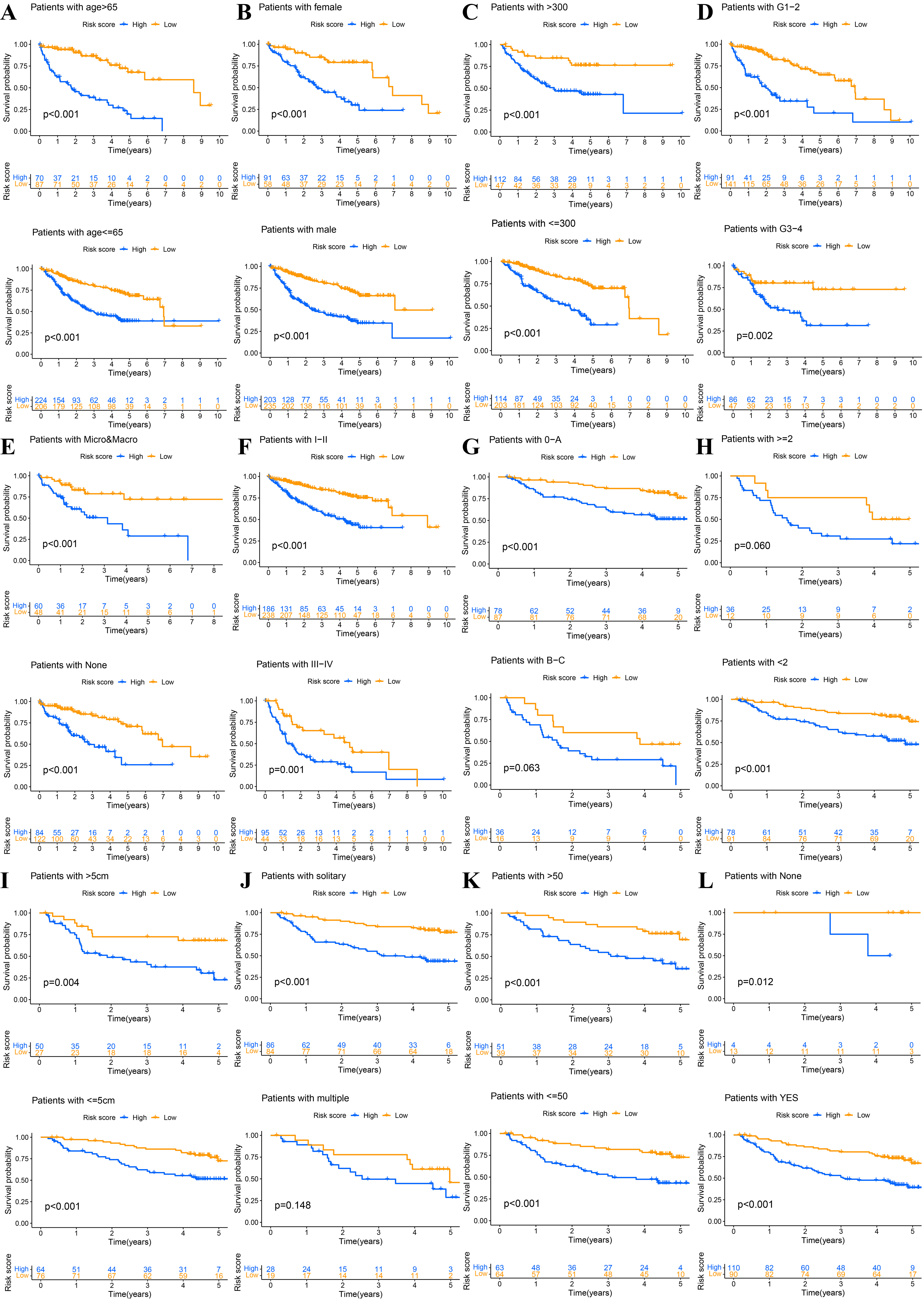

Supplement: Supplementary file 4 — Figure S4 [file CAM4-12-898-s007.jpg]

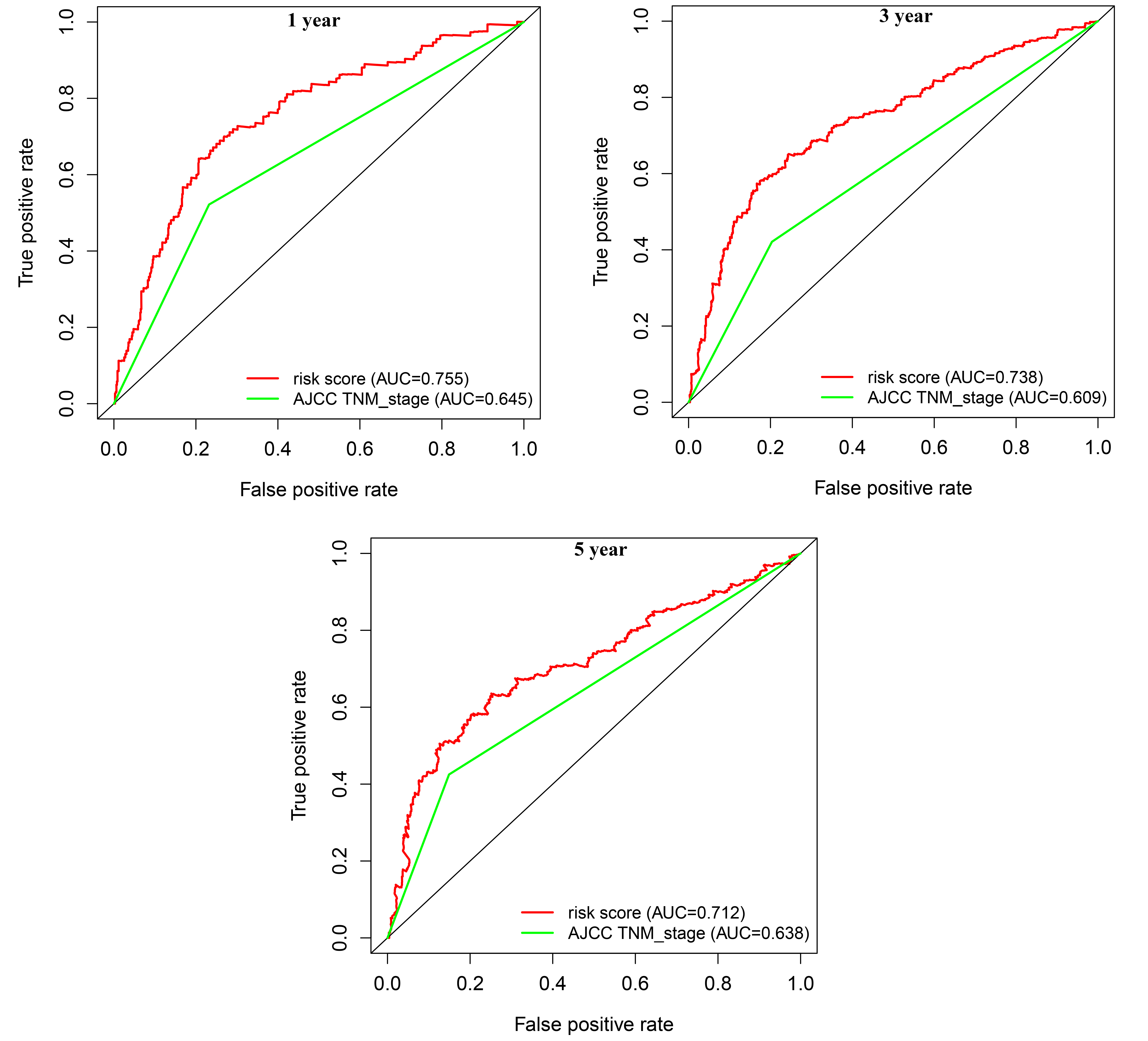

Supplement: Supplementary file 5 — Figure S5 [file CAM4-12-898-s005.jpg]

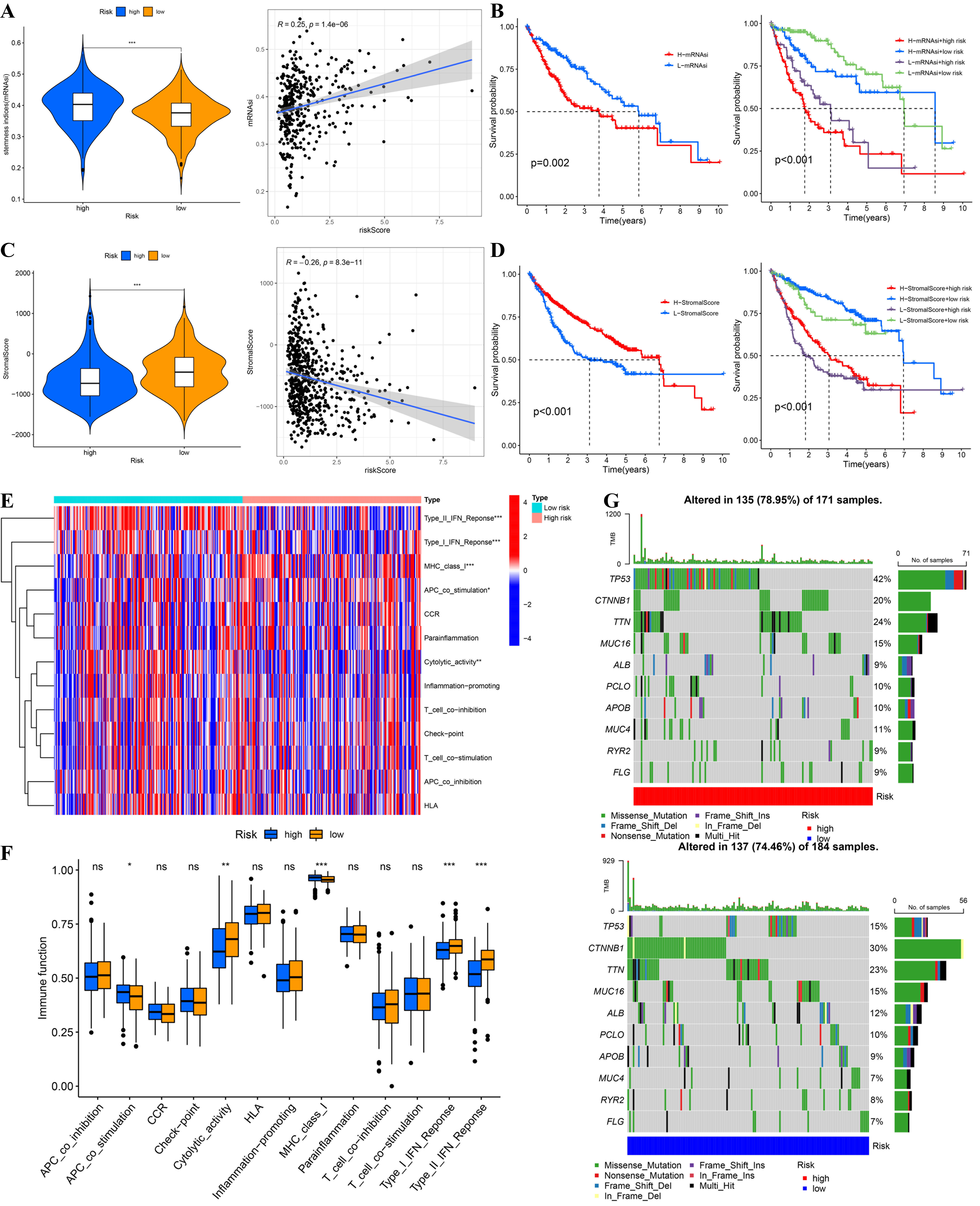

Supplement: Supplementary file 6 — Figure S6 [file CAM4-12-898-s004.jpg]
